# Biological treatment of hazardous heavy metals by *Streptomyces rochei* ANH for sustainable water management in agriculture

**DOI:** 10.1038/s41598-021-88843-y

**Published:** 2021-04-29

**Authors:** Amira M. Hamdan, Heba Abd-El-Mageed, Nevine Ghanem

**Affiliations:** 1 Oceanography Department, Faculty of Science, Alexandria University, Moharam Beih, Anfoushy, Alexandria, 21511 Egypt; 2 Botany and Microbiology Department, Faculty of Science, Alexandria University, Baghdad Street, Moharam Beih, Alexandria, Egypt

**Keywords:** Microbiology, Environmental sciences

## Abstract

Microbial bioremediation of heavy metals-polluted industrial effluents has been adopted as one of the most effective eco-friendly tool to cope up with the harmful effects of metals. This study was designed to investigate the biosorption potential of marine actinomycetes isolated from the Alexandrian Mediterranean Seacoast, Egypt, with their potential use in metal remediation of industrial effluents. Among the nine marine actinomycetes isolates, *Streptomyces rochei* ANH showed the highest versatile metal resistance capability with MIC values of 125 mg/l for Cr^6+^ and 60 mg/l for both Cd^2+^ and Pb^2+^. Additionally, scanning electron micrographs showed complete disintegration of Cr^6+^-treated biomass compared with the control ones where spores remained intact and connected in long chains. The study also aimed to improve the percentage of Cr^6+^ biosorption by *S. rochei* ANH biomass using the statistical designs of Plackett–Burman and Box-Behnken where up to 85% of Cr^6+^ removal was recorded under the following conditions: pH (5), incubation temperature (30 °C), contact time (3 h), agitation speed (90 rpm), initial Cr^6+^ concentration (50 mg/l) and living biomass concentration (10 mg/ml). The results also showed that the percentage of Cr^6+^ biosorption by *S. rochei* ANH decreased gradually beyond these values. Moreover, the results revealed that the use of the biomass of *S. rochei* ANH is an effective biotechnological agent for the biological treatment of heavy metal-contaminated tannery effluent where the percentages of metal removal were in the following order: Ni^2+^ (100%) ≥ Cu^2+^  ≥ Mn^2+^  ≥ Fe^2+^  > Pb^2+^ (95%) ≥ Cd^2+^  > Cr^6+^ (86%). Furthermore, the treated effluent exhibited a stimulating effect on the germination process of *Lepidium sativum* seeds. Therefore, the present study implies that *S. rochei* ANH can be considered a powerful candidate to mitigate hazardous heavy metals pollution from industrial effluents and improve the water quality for agricultural purposes.

## Introduction

The tremendous increase of anthropogenic activities, industrialization, and urbanization, which is related to the industrial revolution taking place during the past decades, is considered a serious threat to public health. Direct disposal of toxic heavy metals, such as cadmium (Cd), chromium (Cr), and lead (Pb), represents one of the most significant hazardous environmental problem that has been ravaging nature and led to deleterious effects on different ecosystems^1,2^. Such a remarkable rise in the environmental burden due to water pollution and scarcity necessitates the sequestration of heavy metals from the industrial wastewater^3,4^.

Various techniques have been designed to ensure the efficient treatment of contaminated waters against the consequences of the indiscriminate release of heavy metals in water bodies. Hence, several treatment technologies, such as chemical precipitation, oxidation/reduction, ion exchange, membrane filtration, and evaporation, were developed to cope with the accumulation of heavy metals in contaminated sites^5–8^. However, conventional methods for treating toxic heavy metals in industrial wastewater are considered inefficient and relatively expensive^9^. Therefore, these shortcomings have led to the use of microorganisms, such as bacteria, yeast, algae, and fungi, in the bioremediation of heavy metals for the promotion of efficient, economically feasible, and eco-friendly alternative technology to mitigate heavy metal concentrations in industrial wastewater to environmentally acceptable levels^10–13^. Several studies have documented the efficient use of *Escherichia coli*, *Neopestalotiopsis clavispora*, *Beauveria bassiana,* and *Metarhizium anisopliae* as cost beneficial and efficient biosorbents for Pb^2+^, Zn^2+^, and Cd^2+^ from aqueous solutions^14–16^ (Table S1).

Chromium is the 7th most abundant element on Earth that occurs mainly in two forms: trivalent (Cr^3+^) and hexavalent (Cr^6+^), where hexavalent chromium compounds exhibit 1000-fold more toxic, mutagenic, and carcinogenic traits than trivalent chromium in biological systems^17,18^. Chromium is extensively used in several industries, like leather tanning, electroplating, wood preservation, pigment fabrication, etc., that are released into natural water resources either directly or indirectly, raising major concerns of chromium-associated environmental pollution^19^.

Technologies employed for the removal of chromium from tannery wastewater produce undesirable chemical by-products and are costly^20^. On the other hand, bioremediation of toxic chromium from the industrial wastewater increased the awareness of the role of microorganisms including *Rhizobium*, *Bacillus*, *Pseudomonas aeruginosa*, *Escherichia coli*, *Vibrio harveyi*, *Alcaligenes*, *Enterobacter*, *Phanerochaete chrysosporium,* and *Shewanella* as an effective strategy to improve the quality of the effluent^21–25^. In this context, actinomycetes, one of the most diverse Gram-positive filamentous bacteria, are characterized by their ability to produce diverse secondary metabolites of immense biotechnological importance besides their efficient usage in the biological treatment of toxic heavy metals from wastewater^26–29^.

As a result, this study was conducted to evaluate the biosorption of toxic hexavalent chromium from aqueous solutions by a novel marine actinomycetes isolate, *S. rochei*, collected from the Alexandrian Mediterranean Seacoast, Egypt. Moreover, there have been attempts to achieve the optimal conditions for maximum chromium removal by *S. rochei* using both Plackett-Burman and Box-Behnken statistical designs. Furthermore, the study was extended to assess the efficacy of *S. rochei* in the biological treatment of a tannery waste effluent to alleviate its environmental impact and evaluate the feasibility of such a treatment method on the germination of *Lepidium sativum* seeds.

## Results and discussion

### Isolation of heavy metal-resistant marine actinomycetes

Nine colonies of actinomycetes, morphologically distinct from sediment samples, were isolated and explored by the agar well diffusion method for the highest MIC levels against hazardous heavy metals, Cr^6+^, Cd^2+^, and Pb^2+^. From the recovered isolates, only one strain, designated as ANH, exhibited the highest multiple resistance capability against the three tested heavy metals with MIC values of 125 mg/l for Cr^6+^ and 60 mg/l for both Cd^2+^ and Pb^2+^ (data not shown). Therefore, strain ANH was selected as a promising candidate for further investigations.

### Identification of ANH actinomycete isolate

**Figure 1:**
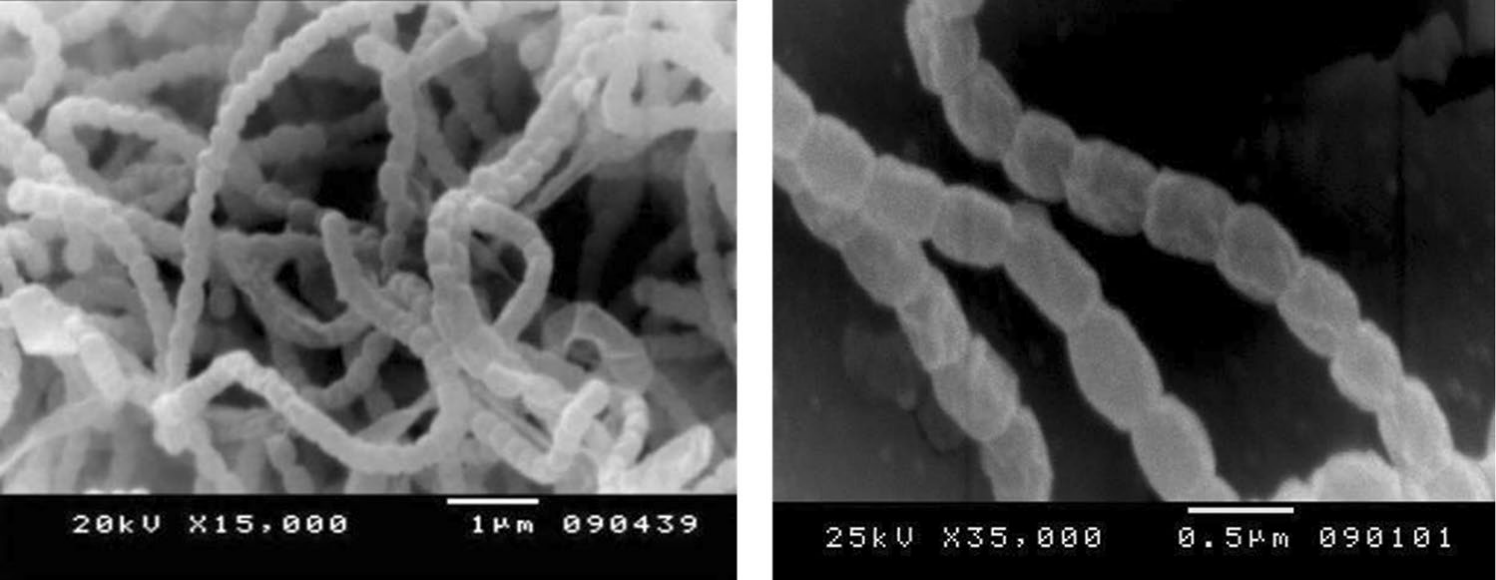
SEM micrographs showing the spore-chain morphology of the marine Actinomycete isolate *S. rochei* ANH.

Scanning electron micrographs of ANH strain revealed that the isolate had smooth-surface cylindrical-shaped spores arranged in spiral long chains (Fig. 1). Accordingly, the morphological characteristics of ANH are consistent with members of the genus *Streptomyces* described by Saurav and Kannabiran^30^ and Hozzein et al.^26^. Furthermore, the biochemical properties of ANH showed that the isolate could utilize D-glucose, cellobiose, and glycerol as sole carbon sources; however, D-xylose and citrate could not be used. Also, the ANH strain displayed catalase, gelatinase, amylase, caseinase, lipase, and urease activities besides H_2_S production, where NO_3_ reduction was negative.

In addition, the results of 16S rRNA gene sequencing confirmed that the selected ANH actinomycete isolate belongs to the genus *Streptomyces* with 99.5% similarity to the *Streptomyces rochei* strain UAE1-3 (accession number MN795133.1) in the GenBank database. According to the phylogenetic comparison, the isolate was identified as *Streptomyces rochei* ANH and the sequences were submitted to the DDBJ under accession no. of LC537844. Lakshmipathy et al.^31^ reported that *Streptomyces* species is the most widely documented genus among other genera in the marine sediments. Moreover, Öztürk^32^ and Bakran et al.^33^ mentioned that species of the genus *Streptomyces* inherit heavy metals resistance genes, allowing them to occupy contaminated environments.

### Antibiotic sensitivity profile of *S. rochei* ANH

The susceptibility of *S. rochei* ANH to various antibiotics was detected using the disc diffusion technique where *S. rochei* ANH exhibited resistance against six tested antibiotics (Imipenem, Piperacillin/tazobactam, Rifamycin, Piperacillin, Cefuroxime, and Levofloxacin) and sensitivity to the other four tested antibiotics (Tobramycin, Cefotaxime, Erythromycin, and Cefaclor) with MAR index value of 0.6 (Table 1). Jain *et al*.^34^ reported that the MAR index reflects the spread of antibiotic resistance among a given population where MAR indices greater than 0.2 indicate that such bacterial strains acquired resistance from their environments due to the excessive use of antibiotics, thus transferring antibiotic resistance genes across those species horizontally. Furthermore, Remenár et al.^35^ indicated that both antibiotic and heavy metals resistance genes are often harbored on the same bacterial plasmids or transposons. On the other hand, Tomova et al.^36^ demonstrated that combing both capabilities of multiple resistances to antibiotics and heavy metals indicates a competitive advantage of the bacterial isolate to adapt to extreme environments and could serve as a basis for their use in bioremediation approaches. Consequently, the high metal resistance capability of these strains may suggest that they can overproduce some multidrug resistance efflux pumps that are known to be involved in bacterial resistance to a wide range of toxic compounds.

**Table 1:** Antibiotic resistance profile of *S. rochei* ANH.

Antibiotics		Code	Inhibition zone (mm)
Cefotaxime (30 μg)		CTX 30	7
Cefuroxime (30 μg)		CXM 30	0
Cefaclor (30 μg)		CEC 30	27
Erythromycin (15 μg)		E 15	8
Imipenem (10 μg)		IPM 10	0
Levofloxacin (5 μg)		LEV 5	0
Piperacillin/Tazobactam (110 μg)		TPZ 110	0
Piperacillin (100 μg)		PRL 100	0
Rifampicin (30 μg)		RF 30	0
Tobramycin (10 μg)		TOB 10	22

### Biosorption of Cr^6+^ by *S. rochei* ANH

**Figure 2:**
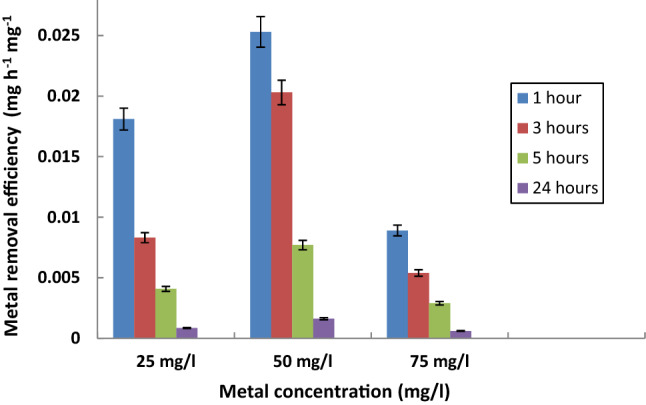
Effect of initial Cr^6+^ concentration on the metal removal efficiency by *S. rochei* ANH at different time intervals. Bars represent standard deviation (SD).

The biomass of *S. rochei* ANH was used to evaluate Cr^6+^-removing potential at different time intervals from 1 to 24 hrs. As shown in Fig. 2, the efficiency of Cr^6+^ removal by *S. rochei* ANH was concentration dependent, where Maximum Removal Efficiencies (MRE) 0.0253 mg h^-1^ mg^-1^ and 0.0203 mg/h/mg were recorded with the increase of the initial metal concentration to 50 mg/l after 1 hr and 3 h, respectively. Afterward, a substantial decrease in Cr^6+^ removal efficiency (0.00061 mg/h/mg) was reported with a further increase in the concentration to 75 mg/l after 24 h.

Several studies documented that heavy metal binding capacity is a physico-chemical reaction where metals rapidly adsorb on the cell surface based on the negatively-charged sites on the cell wall of Gram-positive bacteria^36–39^. Meanwhile, El-Gendy and El-Bondkly^40^ reported that the relatively thick peptidoglycan layer and high phosphorus content in the cell wall of Gram-positive bacteria make actinomycetes more superior metal biosorbents than Gram‐negative bacteria, consequently displaying more electrostatic interactions with heavy metals.

Moreover, it is noteworthy that the initial metal concentration plays a significant role in the metal biosorption and the higher biosorption efficiency of *S. rochei* ANH, where the increase in Cr^6+^ concentration could be attributed to the greater availability of the interaction between the metal ions and the biosorbent as mentioned by Karakagh et al.^41^. Besides, the considerable decrease in the removal efficiency of *S. rochei* ANH with the increase in Cr^6+^ concentration to 75 mg/l might be due to the saturation of the adsorption sites and the lack of sufficient free binding sites for metal biosorption^42^.

### Assessment of Cr^6+^ biosorption by SEM/EDX

**Figure 3:**
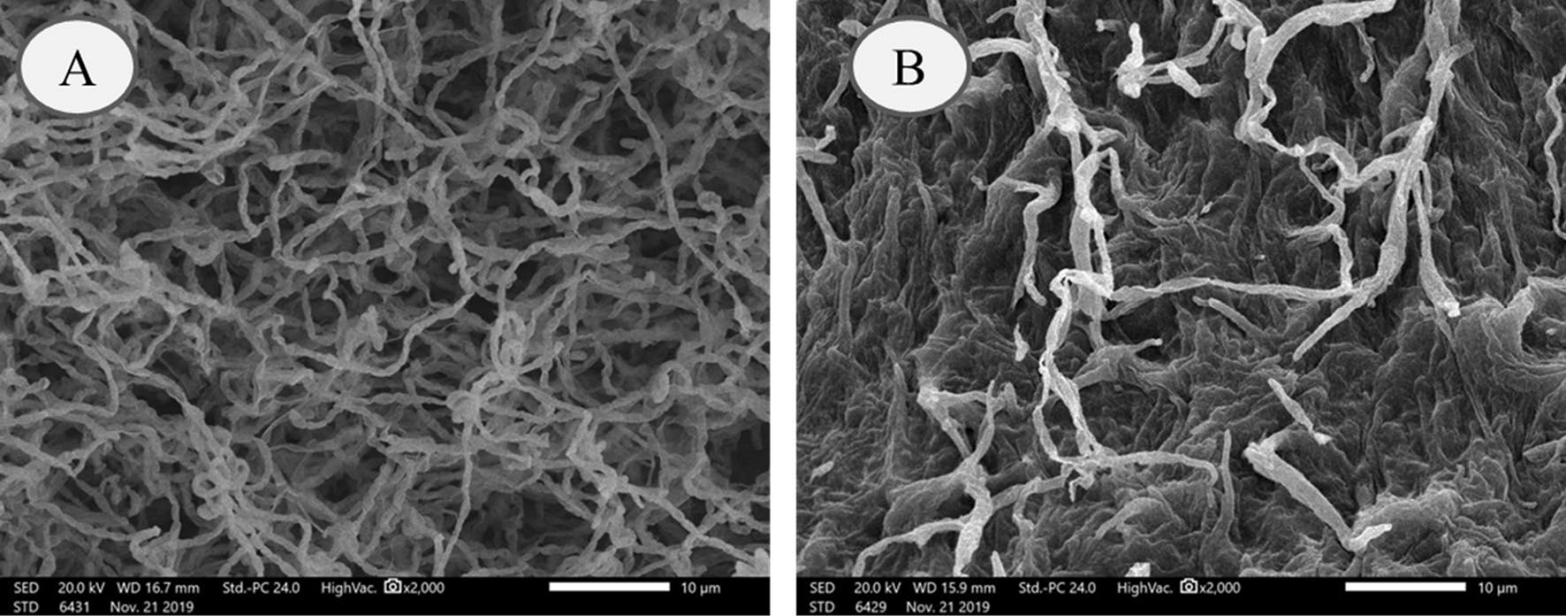
SEM micrographs of the biomass of *S. rochei* ANH **(A)** before and **(B)** after Cr^6+^ biosorption (scale bar represents 10 μm).

SEM micrographs of *S. rochei* ANH before and after Cr^6+^ exposure were conducted to locate morphological alterations in its biomass. The results of SEM analysis corroborated the detrimental effect of loading Cr^6+^ on the biomass of *S. rochei* ANH. As shown in Fig. 3, Cr^6+^-treated biomass was completely distorted and disintegrated compared with the control biomass where spores remained intact, smooth, and connected in long chains. This is in accordance with the findings of Chaudhary et al.^43^, Feng et al.^44^, and Zhenggang et al.^45^ who reported that, under metal stress conditions, the deterioration and disruption in the cellular morphology may result from the interaction forces between metallic ions and surface-active components of the biosorbent.

**Figure 4:**
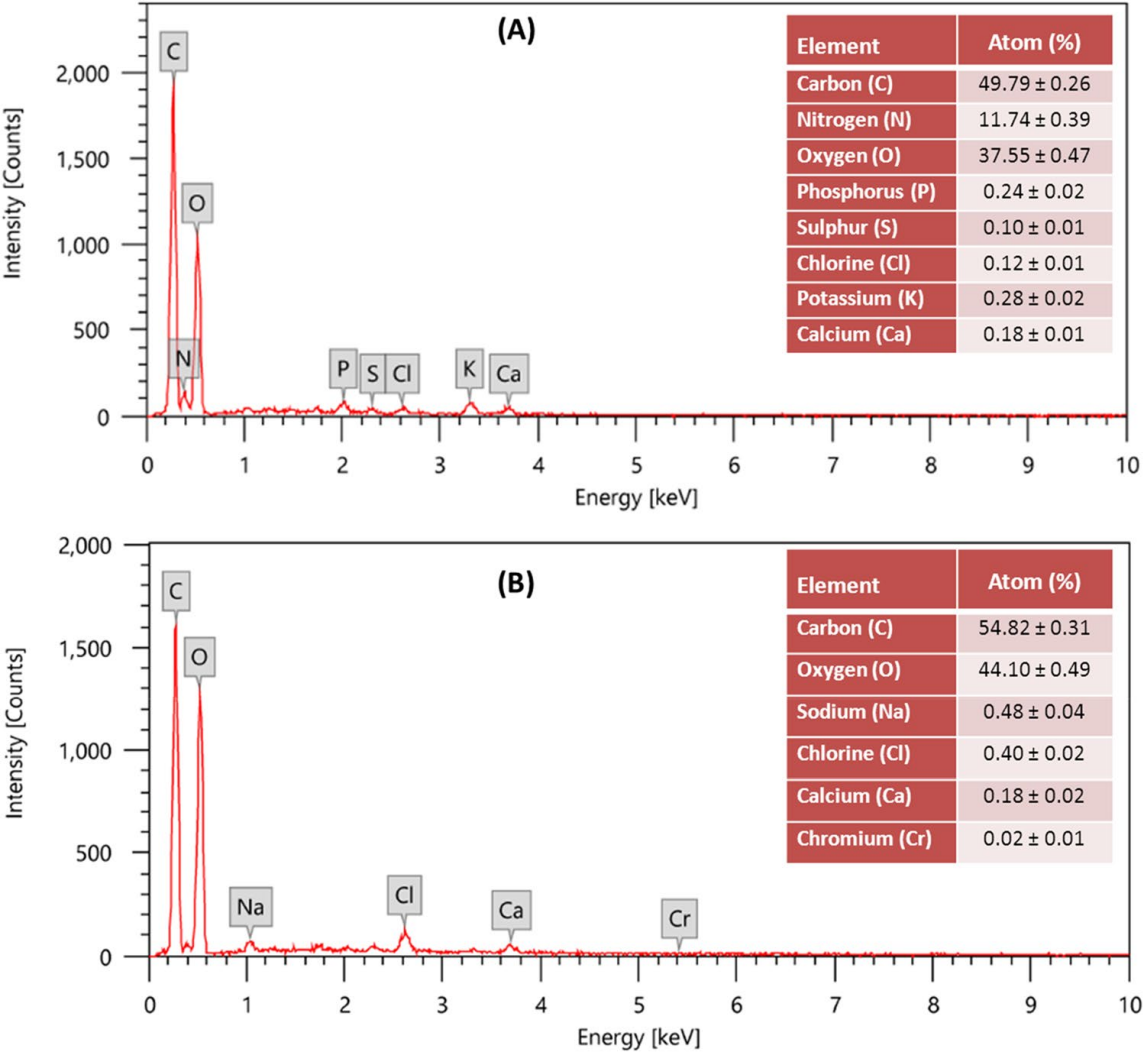
EDX spectra of *S. rochei* ANH control biomass **(A)** and Cr^6+^-treated biomass **(B)**.

EDX analysis was conducted to provide information regarding the elemental composition of the biomass of *S. rochei* ANH and confirm the adsorption of Cr^6+^ ions (Fig. 4). EDX spectra detected the presence of a characteristic signal of Cr on the surface of Cr^6+^-treated biomass at 5.4 keV, where no Cr signal was detected in the control biomass. Moreover, after Cr^6+^ uptake, the analysis of EDX spectra showed the disappearance of N, P, S, and K peaks with the increase in the atomic percentage (At %) of C and O from 49.79 to 54.82 and 37.55 to 44.10, respectively, indicating a possible competitive exchange of Cr^6+^ with the functional ion groups on the cell surface. This concedes with the findings of Xia et al.^46^ and Jin et al.^47^ who proved that the ion-exchange mechanism is a crucial part or even a dominant one in the biosorption process of metal ions.

### Evaluation of significant variables affecting Cr^6+^ biosorption by *S. rochei* ANH

**Figure 5:**
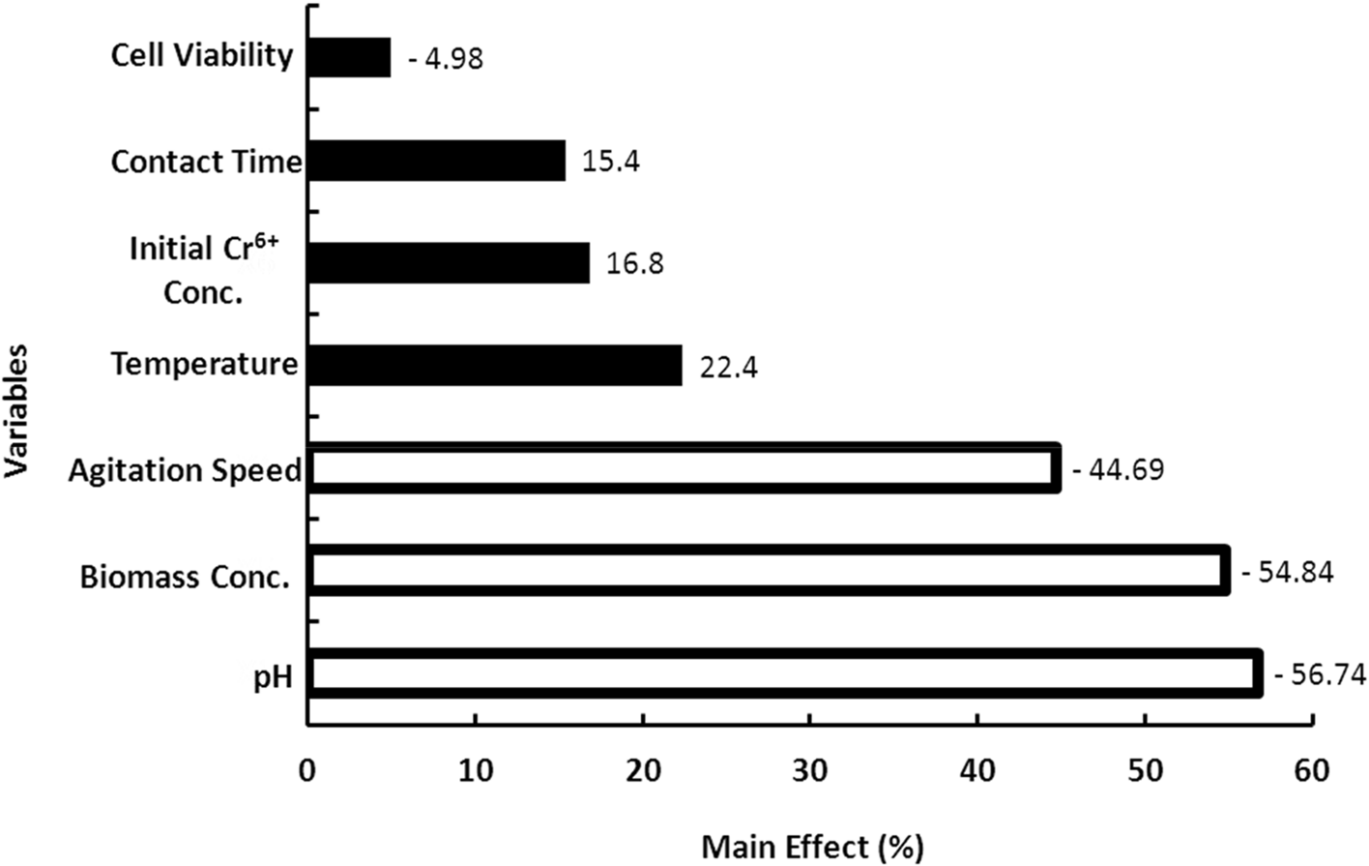
Pareto chart representing the percentage main effects of the different independent variables affecting the biosorption of Cr(VI) by *S. rochei* ANH using PBD. The white bars represent the significant variables, while the black bars represent the insignificant variables.

Plackett-Burman Design (PBD) was conducted to identify the key variables that significantly influence the percentage removal of Cr^6+^ by *S. rochei* ANH as depicted in Table 2. The Pareto chart (Fig. 5) presented the percentage of the main effect of each investigated variable that impacted the biosorption characteristics in the following order: X_1_ > X_5_ > X_4_ > X_2_ > X_6_ > X_3_ > X_7_. The results showed that pH, biomass concentration, and agitation speed had significant negative effects on Cr^6+^ biosorption with confidence levels above 95% (*P* < 0.05). The other tested variables, incubation temperature, contact time, initial metal concentration, and cell viability had insignificant effects (*P* > 0.05) on metal removal.

**Table 2:** Plackett–Burman design for the evaluation of significant variables affecting Cr^6+^ removal by *S. rochei* ANH.

Trials	X_1_	X_2_	X_3_	X_4_	X_5_	X_6_	X_7_	Cr^6+^ removal (%)
1	+ 1	-1	-1	+ 1	-1	+ 1	+ 1	15.30
2	+ 1	+ 1	-1	-1	+ 1	-1	+ 1	13.10
3	+ 1	+ 1	+ 1	-1	-1	+ 1	-1	41.80
4	-1	+ 1	+ 1	+ 1	-1	-1	+ 1	42.00
5	+ 1	-1	+ 1	+ 1	+ 1	-1	-1	0
6	-1	+ 1	-1	+ 1	+ 1	+ 1	-1	25.50
7	-1	-1	+ 1	-1	+ 1	+ 1	+ 1	38.70
8	-1	-1	-1	-1	-1	-1	-1	47.40

Subsequently, the significant variables, i.e., pH, biomass concentration, and agitation speed, identified by PBD were evaluated for the next stage in the optimization of Cr^6+^ biosorption by *S. rochei* ANH using the BBD technique. The relationship between the independent variables and the response value was expressed in the following polynomial equation:

$$ {\text{Cr}}^{{6 + }} {\text{ biosorption}} (\% ) = 46{.8 + 14}{\text{.5 X}}_{{1 }} - {1}{\text{.4 X}}_{{2 }} { + 3}{\text{.3 X}}_{{3 }} - {30}{\text{.3 X}}_{{1}}^{{2}} { + 11}{\text{.6}} {\text{X}}_{{2}}^{{2}} + {11}{\text{.4 X}}_{{3}}^{{2}} { + 0}{\text{.6 X}}_{{1 }} {\text{X}}_{{2 }} - {4}{\text{.2 X}}_{{1 }} {\text{X}}_{{3 }} { + 10}{\text{.9 X}}_{{2 }} {\text{X}}_{{3 }}  $$

**Figure 6:**
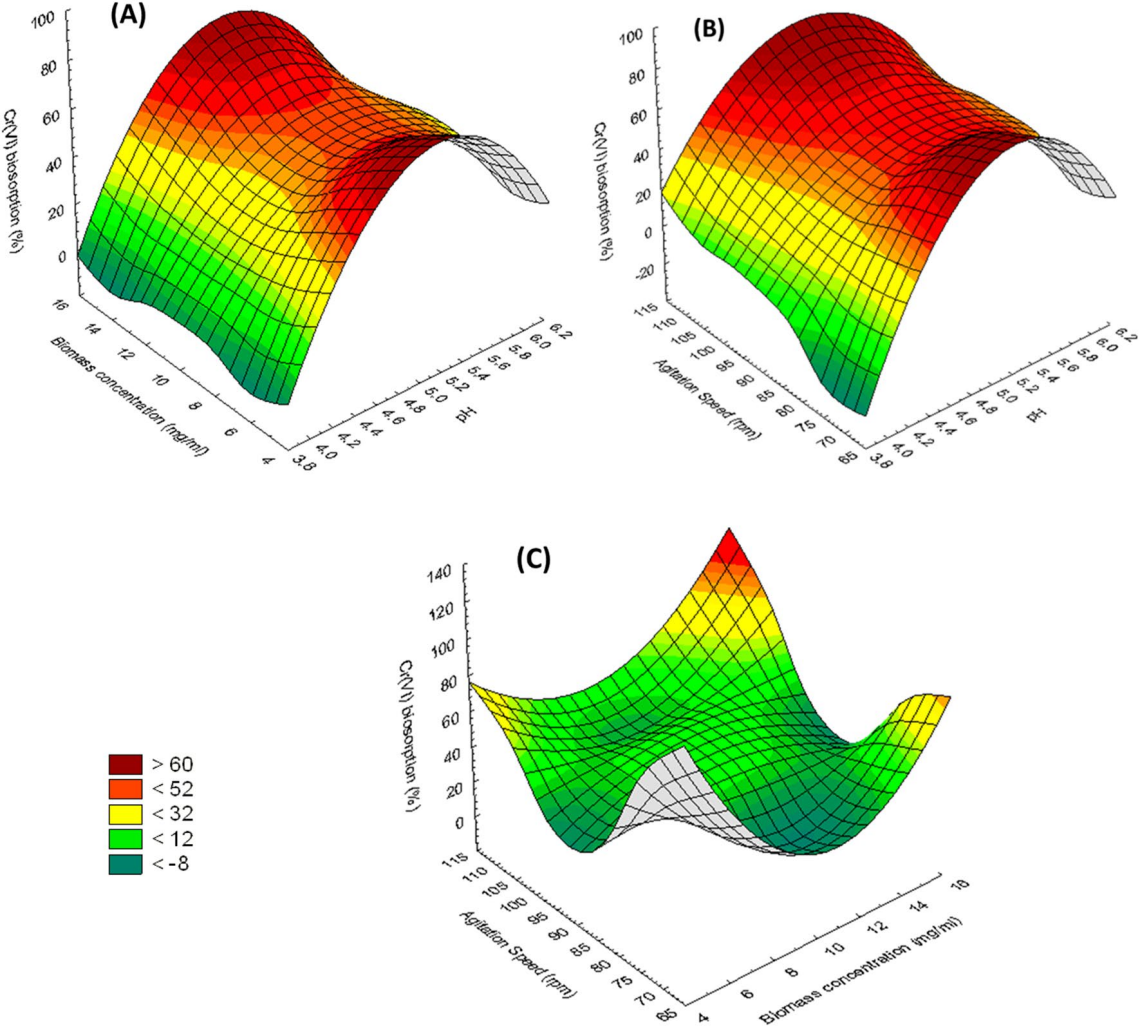
3-D surface plots showing the effect of the interaction between **(A)** pH and biomass concentration (mg/ml), **(B)** pH and agitation speed (rpm) and **(C)** biomass concentration (mg/ml) and agitation speed (rpm), on Cr^6+^ biosorption (%) by *S. rochei* ANH.

Moreover, the 3D response surface plots graphically demonstrated the interaction of the tested variables on the biosorption of Cr^6+^ (Fig. 6). The results showed that the percentage of Cr^6+^ biosorption by *S. rochei* ANH consistently increased (~ 80%) with the increase in pH, biomass concentration, and agitation speed from 4 to 5, 5 to 10 mg/ml, and 70 to 90 rpm, respectively. Beyond these values, the percentage of Cr^6+^ removal decreased gradually.

The results of the present study are in accordance with previous studies demonstrating that heavy metal removal by actinomycetes is markedly affected by changing the pH of the solution, where the maximum metal uptake was recorded at slightly acidic or neutral pH values and drastically decreased in either extreme pH ranges^48,49^. Saurav and Kannabiran^30^ and Jin et al.^47^ reported that the increased biosorption at low pH may be related to an increase in the negatively charged groups on the surface of the biomass which favored the electrostatic attraction with metal cations. On the contrary, at higher pH values, the biosorption efficiency of metal ions may be negligible due to the precipitation of metal ions or the competition of H^+^ ions with metal ions on the active sites and subsequently hinders the adsorption process.

Moreover, regarding the effect of biomass concentration on metal removal, Al-Kadeeb and Al-Rokban^48^ revealed that the rate of biosorption of aluminum by *S. albus* and *S. diastaticus* reached its maximum values with lower biomass dosage, whereas reduced metal removal at higher biomass dosage may be due to a possible "screen effect" of the dense layer of cells that protects the biomass active sites from binding metal ions. On the other hand, Raaman et al.^20^ and Xia et al.^46^ demonstrated that at higher biomass concentration, agglomeration of cells occurs with a consequent reduction in the active sites on the biomass that impedes their metal removal efficiency. However, in contrast, Abd El-Motaleb et al.^50^ reported that the sorption efficiency of *S. cyaneus* Kw 42 increased with the increase in the biosorbent dosage where more binding sites were available, leading to the complete removal of metal ions from the aqueous solution.

For agitation speed, Batool and Hasnain^51^ indicated that the biosorption of Cr^6+^ is highly affected by the speed of agitation where moderate speeds (100–120 rpm) facilitate proper contact between the biomass and the metal ions in the aqueous solution compared with static conditions. Furthermore, a decrease in metal removal efficiency at higher speeds (up to 500 mg/l) may be a consequence of the vortex phenomenon attributed to the reduction of the contact between biomass active sites and metal ions^52^.

### Biological treatment of the tannery wastewater by *S. rochei* ANH

Table 3 shows the analysis of the heavy metal content in the collected tannery effluent where metal values exceeded the standard limits recommended by the United States Environmental Protection Agency (USEPA) guidelines^53^.

**Table 3:** The average concentration of heavy metals in the tannery wastewater sample before treatment.

Metals		Initial concentration in the tannery effluent (mg/l)	Concentration in the US EPA guidelines (mg/l)
Chromium (Cr)		225	0.1
Cadmium (Cd)		15.4	0.1
Copper (Cu)		2.8	1.0
Lead (Pb)		14.5	0.2
Nickel (Ni)		6.5	0.1
Iron (Fe)		10.5	0.3
Manganese (Mn)		2.5	1.0

The potency of *S. rochei* ANH to facilitate the biological treatment of toxic heavy metals in tannery wastewater was examined. The percentages of metal removal by *S. rochei* ANH were in the following descending order: Ni^2+^ ≥ Cu^2+^ ≥ Mn^2+^ ≥ Fe^2+^ > Pb^2+^ ≥ Cd^2+^ > Cr^6+^, where the used biomass achieved 100% removal of Ni^2+^, Cu^2+^, Mn^2+^ and Fe^2+^, followed by 95% removal of Pb^2+^ and Cd^2+^, and, finally, 86% removal of Cr^2+^ ions from the industrial effluent within 3 h.

Similar investigations were carried out by El-Gendy and El-Bondkly^40^ who reported that the dead biomass of actinomycetes biosorbents *Nocardiopsis* sp. MORSY1948 and *Nocardia* sp. MORSY2014 exhibited the highest affinity to remove Cr^6+^ (95.22%) > Ni^2+^ (93.53%) > Zn^2+^ (90.37%) that could be present in real industrial wastewater. Abd El-Motaleb et al.^50^ reported that the dead biomass of *Streptomyces cyaneus* Kw42 resulted in the complete removal of Cu^2+^, Pb^2+^, and Cd^2+^ ions from wastewater samples. Moreover, Ameen *et al*.^13^ indicated that using the biosorbent packed in a dialysis tube effectively facilitates the collection of cells from the treated industrial effluent for further use.

### Phytotoxicity assessment of the treated tannery effluent

Seeds of *Lepidium sativum* were used to detect the inhibitory effect of the biologically treated tannery effluent. As shown in Table 4, the untreated tannery effluent had a deleterious effect of 70% on the germination potential of seeds, while seeds exposed to biologically treated effluent showed a high germination percentage (90%), indicating that the treated effluent can be considered free of phytotoxin (Fig. S1). On the other hand, the value of RRG exceeded 100%, which indicates a stimulating effect of the biologically treated effluent on root growth and the improvement of the root elongation compared with the control set, as reported by Pampuro et al.^54^. In this context, Pavel et al.^55^ indicated that at low concentrations (~25 mg/l), metal ions behave as micronutrients with a consequent stimulating impact on the germination process.

**Table 4:** The influence of the tannery effluent on the germination of *Lepidium sativum* seeds.

Trial	RSG (%)	RRG (%)	GI (%)
Untreated tannery effluent	30	45	13.5
Biologically-treated tannery effluent	90	135	121.5

## Materials and methods

### Sample collection and isolation of marine actinomycetes

Twenty-five sediment samples were aseptically collected from different sites along the seacoast of Alexandria, Egypt, in sterile screw-cap falcon tubes and stored at 5°C until further transportation to the laboratory. Samples were dried at 30°C for 24 hrs, treated with CaCO_3_ (1:1 w/w), and incubated at 30 °C for 3 days. Isolation of marine actinomycetes from the sediment samples was carried out by the spread plate technique^26^ on an International Streptomyces Project 2 (ISP2) agar medium containing (g/l): malt extract, 10.0; yeast extract, 4.0; dextrose, 4.0; agar, 20.0, supplemented with filter-sterilized Nystatin (50 μg/ml) and Nalidixidic acid (20 μg/ml) to inhibit the growth of fungal and bacterial contaminants, respectively^56^. Then, the plates were incubated aerobically at 30 °C for up to 14 days, and actinomycetes colonies—with their characteristic powdery appearance—were picked up and maintained in ISP2 agar slants at 4 °C for routine use while being stored in 20% (v/v) glycerol solution at – 80 °C^57^.

### Screening for heavy metal-resistant marine actinomycetes

Standard solutions of Pb(NO_3_)_2_, Cd(NO_3_)_2_, and Cr(NO_3_)_3_ of 1000 mg/l were purchased from CHEM-LAB (Belgium). Solutions were sterilized by filtration through 0.45μm Millipore bacterial filters (Advantec, Co. Ltd. Tokyo, Japan) and used for the preliminary screening of heavy metal-resistant actinomycetes isolates by the agar well diffusion method^35^. Briefly, wells (8 mm diameter) were punched in ISP2 agar media, seeded with each actinomycete isolate via sterile borers. To each well, 100 μl of the standard metal solution, at various concentrations (30–500 mg/l), was loaded and incubated at 30 °C for 7 days. The diameter of growth inhibition around each well was measured (in millimeters) and the Minimum Inhibitory Concentration (MIC) of each metal, that prevents visible growth of actinomycetes, was recorded^13^. The actinomycete isolate with the highest metal-resistance ability was selected for further studies.

### Characterization of the potential marine actinomycete isolate

Spore chain morphology and surface ornamentation of the prominent metal-resistant actinomycete isolate were observed under a Scanning Electron Microscope (SEM) according to Li et al.^58^. Biochemical characterization of the potential isolate was performed using the following tests: catalase reaction, NO_3_ reduction, H_2_S production, hydrolysis of starch, casein, gelatin, Tween 80 and urea and assimilation of D-glucose, glycerol, cellobiose, D-xylose, and citrate as a sole carbon source^59^. Moreover, genomic DNA extraction and 16S rRNA gene sequencing of the selected isolate were applied in accordance with the method described by El-Naggar et al.^60^. Similarity search was performed in the GenBank database at the NCBI using the online BLAST program (http://blast.ncbi.nlm.nih.gov/Blast.cgi) and deposited in the DNA Data Base of Japan (DDBJ).

### Antibiotic susceptibility test

The antibiotic resistance of the marine actinomycete isolate was tested against 10 different commercial antibiotic discs; Erythromycin (15 μg), Cefaclor (30 μg), Imipenem (10 μg), Cefotaxime (30 μg), Piperacillin/ tazobactam (110 μg), Rifamycin (30 μg), Piperacillin (100 μg), Cefuroxime (30 μg), Levofloxacin (5 μg) and Tobramycin (10 μg) (Oxoid, UK) using the disc diffusion method^61^. Antibiotic discs were aseptically placed on the surface of the ISP2 agar plates inoculated with the actinomycete isolate and incubated at 30 °C for 7 days. The antibiotic resistance profile of the actinomycete isolate was assessed according to the measured diameter (mm) of the inhibition zone around each antibiotic disc and the Multiple Antibiotic Resistance (MAR) index was calculated according to Ameen et al.^13^:

$$ {\text{MAR index}} = \frac{{\text{a}}}{{\text{b}}} $$

where "a" represents the number of antibiotics to which the actinomycete isolate is resistant and "b" the total number of antibiotics used.

### Biosorption of Cr (VI) by marine actinomycete isolate

The biomass of the actinomycete isolate was prepared following Latha et al.^42^. Cells were cultivated in 2 l of the ISP2 medium in a rotary shaker at 120 rpm for 7 days at 30 °C. Then, the biomass was harvested by centrifugation at 6000 rpm for 10 min and washed three times with sterile ultra-pure Milli-Q (MQ) water. Subsequently, cell pellets (30 mg/ml) were re-suspended in 5 ml sterile MQ water amended with various concentrations (25, 50, and 75 mg/l) of Cr (VI) and incubated at 30 °C for 24 h. Aliquots were collected at different time intervals (1, 3, 5 and 24 h), and centrifuged at 6000 rpm for 10 min. The residual Cr^6+^ concentration in the supernatant was measured by an atomic absorption spectrophotometer (Perkin Elmer-2380, USA)^30^. All experiments were conducted in triplicates and the mean values were used to calculate the uptake capacity (*q*, mg/mg) and the removal efficiency (MRE, mg/h/mg) of Cr^6+^ according to the following formula^47^:

$$ {\text{q }}= \frac{{{\text{C}}_{{\text{i}}} {\text{ - C}}_{{\text{f}}} }}{{\text{W}}}\times{\text{  V}} $$

where "C_*i*_" and "C_*f*_" are the initial and final concentrations of Cr^6+^ (mg/l), respectively, "V" is the reaction volume (ml) and "W" is the total cell biomass (mg) used in the reaction mixture.

$$ {\text{MRE }} =\frac{{{\text{C}}_{{\text{i}}} {\text{ - C}}_{{\text{f}}} }}{{\left( {{\text{t}}_{{\text{f }}} {\text{ - t}}_{{\text{i}}} } \right){\text{ W}}}} $$

where "t_*i*_" and "t_*f*_" are the initial and final contact times, respectively.

### Scanning electron microscope (SEM) and energy dispersive X-ray analysis (EDX)

SEM analysis was used to evaluate the morphological changes of actinomycete isolates in response to Cr^6+^ biosorption. The collected cell pellets of both Cr-free (control) and Cr-treated samples were fixed with 2.5% (v/v) aqueous glutaraldehyde for 2 h and processed according to Ameen et al.^13^ for the examination using SEM (JEOL JSM 5400 LV, japan) equipped with EDX (JEOL JSM 6360 LA, Japan) for the detection of the metal composition of the biomass surface^39^.

### Optimization of Cr^6+^ biosorption process using response surface methodology (RSM)

Biosorption experiments were conducted using sequential statistically designed experiments composed of Plackett–Burman design (PBD)^62^ followed by Box–Behnken design (BBD)^63^.

### Plackett–Burman design (PBD)

PBD was performed to identify the important variables that have a significant effect on Cr^6+^ biosorption by the marine actinomycete isolate. Seven independent variables were investigated in two levels, low (1) and high (+1) levels, including: pH (X_1_), incubation temperature (X_2_), contact time (X_3_), agitation speed (X_4_), biomass concentration (X_5_), initial metal concentration (X_6_), and cell viability (X_7_) (Table 5). All experiments were conducted in triplicate sets where samples were withdrawn to measure residual Cr^6+^ concentration by an atomic absorption spectrophotometer, and the percentage of Cr^6+^ removal was calculated by the following equation^13^:

$$ {\text{Metal removal }}\left( {{\% }} \right){ = }\frac{{{\text{C}}_{{\text{i}}} {\text{ - C}}_{{\text{f}}} }}{{{\text{C}}_{{\text{i}}} }} \times {\text{ 100}} $$

**Table 5:** Independent variables for the evaluation of Cr^6+^ biosorption by the selected Actinomycete isolate using Plackett–Burman design.

Independent variables	Codes	Experimental levels	
		1	+ 1
Initial pH	X_1_	5	8
Incubation temperature (°C)	X_2_	20	40
Contact time (h)	X_3_	1	5
Agitation speed (rpm)	X_4_	90	150
Biomass concentration (mg/ml)	X_5_	10	50
Initial Cr^6+^ concentration (mg/l)	X_6_	25	75
Cell viability	X_7_	Dead^a^	Live

Where; 1 = low level; + 1 = high level.

^a^Dead cells where the biomass was killed by autoclaving at 121ºC for 20 min.

where "C_*i*_" and "C_*f*_" are the initial and final concentrations of Cr^6+^, respectively.

The effect of each variable on the percentage of Cr^6+^ biosorption was determined based on the following equation^64^:

$$ {\text{Y }}= \beta _{{0}} {{ + \Sigma  }}\beta_{{1}} {\text{X}}_{{1}} $$

where "Y" is the response (Cr^6+^ biosorption, %), "β_0_" the model intercept, "β_1_" the linear coefficient, and "X_1_" the level of the independent variable.

### Box–Behnken design (BBD)

After selecting the significant variables affecting Cr^6+^ biosorption, BBD was carried out to elucidate the optimum biosorption conditions and ascertain the interaction between those independent variables. In triplicate sets, Cr^6+^ biosorption was assessed by three significant variables, namely pH, biomass concentration, and agitation speed in 15 experimental trials at three different levels: low (1), middle (0), and high (+1) as shown in Table 6.

**Table 6:** Box-Behnken significant variables for Cr^6+^ biosorption by actinomycete isolate.

Variables	Codes	1	0	+ 1
pH	X_1_	4	5	6
Biomass concentration (mg/ml)	X_2_	5	10	15
Agitation speed (rpm)	X_3_	70	90	110

The interaction effect between the percentages of Cr^6+^ removal and the significant independent variables were estimated using the following second-order polynomial equation^65^:

$$ {\text{Y  }}= \beta_{{0}} {{ + \beta }}_{{1}} {\text{X}}_{{1 }} {{ + \beta }}_{{2}} {\text{X}}_{{2 }} {{ + \beta }}_{{3}} {\text{X}}_{{3 }} {{ + \beta }}_{{{11}}} {\text{X}}_{{1}}^{{2}} {{ + \beta }}_{{{22}}} {\text{X}}_{{2}}^{{2}} + {\beta }_{{{33}}} {\text{X}}_{{3}}^{{2}} {{ + \beta }}_{{{12}}} {\text{X}}_{{1 }} {\text{X}}_{{2 }} + {\beta }_{{{13}}} {\text{X}}_{{1 }} {\text{X}}_{{3 }} + {\beta }_{{{23}}} {\text{X}}_{{2 }} {\text{X}}_{{3 }} $$

where "Y" is the predicted response (Cr^6+^ biosorption, %) and "β_0_" the regression coefficient. "β_1_", "β_2_", and "β_3_" are the linear coefficients, "β_11_", "β_22_", and "β_33_" the quadratic coefficients, "β_12_", "β_13_", and "β_23_" the interaction coefficients, and "X_1_", "X_2_", and "X_3_" the independent variables.

### Efficiency of the actinomycete isolate in the treatment of a tannery effluent

Effluent samples were collected from the outlet pipes of a leather tannery factory in Alexandria, Egypt, in sterile glass bottles and stored at 5 ºC. Thereafter, 500 ml of wastewater samples were filtered through Whatman No. 1 filter paper to remove suspended partics in the effluent, and the filtrate was analyzed for heavy metals content by an atomic absorption spectrophotometer. Cells of the actinomycete isolate (10 mg/ml) were packed in dialysis tubing and immersed in 100 ml of the tannery effluent for 3 h at 25 °C with an agitation speed of 100 rpm. Subsequently, the treated samples were withdrawn, and the concentration of residual metal ions was measured by an atomic absorption spectrophotometer. Then, the percentage of metal removal was estimated^50^. After biological treatment, the treated effluent was stored at 5 ºC for further toxicity investigations.

### Seed germination test

Toxicity assessment of the treated tannery effluent on the germination of *Lepidium sativum* seeds had been investigated according to Pavel et al.^55^. Accordingly, the germination test was conducted in three sets: untreated tannery effluent, biologically treated effluent, and a control set where distilled water was included. For each set, three Petri dishes (90 × 15 mm) were prepared with three layers of Whatman filter paper No. 1 at the bottom as an absorbent medium for the seeds to grow. The bioassay involves the exposure of 10 seeds of *L. sativum,* suitably distributed in each Petri dish, to 10 ml of the effluent/water samples and covered with a lid. For germination, Petri dishes were incubated in darkness for 72 h at 28 ºC. At the end of the test, the numbers of germinated seeds were counted and the average length of root and stem of the germinated seeds were measured in all treatments. Consequently, the percentage of Seed Germination (SG), Relative Seed Germination (RSG), Relative Root Growth (RRG), and percentage of Germination Index (GI) were expressed using the following equations^66^:

$$ {\text{SG }}\left( {{\% }} \right){ = }\frac{{{\text{No}}{\text{. of germinated seeds}}}}{{{\text{No}}{\text{. of total seeds}}}} \times{\text{  100}} $$

$$ {\text{RSG }}\left( {{\% }} \right){ = }\frac{{{\text{No}}{\text{. of germinated seeds in the effluent - treated set}}}}{{{\text{No}}{\text{. of germinated seeds in the control set }}}} \times {\text{  100}} $$

$$ {\text{RRG }}\left( {{\% }} \right){ = }\frac{{\text{Mean root length of the effluent - treated set}}}{{\text{Mean root length of the control set }}} \times {\text{ 100}} $$

$$ {\text{GI }}\left( {{\% }} \right){ = }\frac{{\text{RSG x RRG}}}{{{100}}} $$

### Statistical analysis of data

All experiments were carried out in triplicates and the results were expressed as the mean ± standard deviation (SD). The obtained data were subjected to a One-Way Analysis of Variance (ANOVA) followed by Student’s *t*-test to estimate *t*-value, *P-*value, and confidence levels. The results were considered statistically significant when *P* < 0.05. All statistics were performed using the Statistical Package for the Social Sciences (SPSS) program (Version 12.0, SPSS Inc., Chicago, IL). The three-dimensional (3D) surface plots were generated using the STATISTICA software (Version 10.0, StatSoft Inc., Tulsa, USA).

## Conclusion

Overall, the present study provides evidence indicating that the marine actinomycete isolate, *S. rochei* ANH, is a powerful agent for the versatile removal of hazardous heavy metals, such as Ni^2+^, Cu^2+^, Pb^2+^, Cd^2+^, and Cr^6+^, in industrial wastewater. The statistical approaches composed of Plackett-Burman and Box-Behnken designs reported that biosorption of Cr^6+^ by *S. rochei* ANH was dependent on the increase of initial pH (5), biomass concentration (10 mg/ml), and agitation speed (90 rpm). Moreover, tannery wastewater treated with the biomass of *S. rochei* ANH was considered freed of phytotoxin, where seeds of *L. sativum* exposed to the biologically treated effluent showed a high germination percentage (90%) compared with those exposed to the untreated effluent. To the best of our knowledge, this is the first research carried out on the marine actinomycetes, *S. rochei* ANH, for eco-friendly treatment of real heavy metal–polluted industrial effluent for the improvement of the wastewater quality and its application in the seed germination process. The results also draw attention to the design and development of bioremediation experiences at heavy metal–polluted areas using the potentially useful actinomycetes strains in the agricultural field.

## Supplementary Information

Supplementary Information.
