# Supplementary material for: Biological treatment of hazardous heavy metals by Streptomyces rochei ANH for sustainable water management in agriculture
Source: Sci Rep. 2021 Apr 29;11:9314. doi: 10.1038/s41598-021-88843-y (PMC8085208; doi:10.1038/s41598-021-88843-y)
Supplement: Supplementary file 1 — Supplementary Information. [file 41598_2021_88843_MOESM1_ESM.docx]

**Table 1S.** Microorganisms used in Cr^6+^ Removal

| **Microorganism** | **Source** | **Cr^6+^ removal**  **(%)** | **Culture condition** | **Reference** |  |
| --- | --- | --- | --- | --- | --- |
| **Actinomycetes** | |  |  |  |  |
| *Streptomyces* sp. CG252 | | Soil, Colombia | 95% | 37°C, 20 days, 100 mg/l (Cr^6+^) | **1** |
| *Streptomyces* sp. | | Mining areas, Morocco | 100% | room temp., 3 hrs, 50 mg/l (C^6+^) | **2** |
| *Streptomyces griseus* | | Soil | 100% | 28°C, 32 hrs, 20 mg/l (Cr^6+^) | **3** |
| *Nocardiopsis halophile, Nocardiopsis rosea* | | Soil, Saudia Arabia | 100% | 30°C, 4 weeks, 17 mg/l (Cr^6+^) | **4** |
| *Streptomyces* MC1 | | Sediments, Argentina | 96% | 7 days, 50 mg/l (Cr^6+^) | **5** |
| **Fungi and yeast** | |  |  |  |  |
| *Aspergillus niger, Aspergillus parasiticus* | | landfill and sludge samples, Nigeria | 96.3%  91.6% | 96 hrs, 20 mg/l (Cr^6+^) | **6** |
| *Aspergillus awamori* | | Waste, Mexico | 78.6% | 40°C, 48 hrs, 100 mg/l (Cr^6+^) | **7** |
| **Bacteria** | |  |  |  |  |
| *Pseudomonas gessardii* LZ-E | | Yellow river, china | 95% | 48 hrs, 10 mg/l (Cr^6+^) | **8** |
| *Rhodobacter sphaeroides* | | ATCC 17023 | 80% | 43 mg/l (Cr^6+^) | **9** |

1- Morales, D.K., Ocampo, W., Zambrano, M.M. Efficient removal of hexavalent chromium by a tolerant *Streptomyces sp.* affected by the toxic effect of metal exposure. *J. Appl. Microbiol*. **130,** 1364-5072 (2007).

2- El Baz, S., Baz, M., Barakate, M., Hassani, L., El Gharmali, A., Imziln, B. Resistance to and accumulation of heavy metals by Actinobacteria isolated from Abandoned Mining Areas*. Sci. World* *J*. **2015,** 2-14 (2015).

3- Laxman, R.S., More, S. Reduction of hexavalent chromium by *Streptomyces griseus*. *Miner. Eng*. **15**, 831-837 (2002).

4- El turk, I.M., Kiki, M.J. Heavy Metals Removal from Raw Industrial Wastewater by Halophilic Actinomycetes Isolated from Saudi Saline Soil. *J. Int. Environ. Appl. Sci*. **6**, 606-612 (2011).

5- Polti, M.A., Amoroso, M.J., Abate, C.M. Intracellular chromium accumulation by *Streptomyces* sp. MC1. *Water Air Soil Pollut*.  **214**, 49–57 (2011).

6- Shugaba, A., Buba, F., Kolo, B.G., Nok, A.J., Ameh, D.A., Lori, J.A. Uptake and reduction of hexavalent chromium by *Aspergillus niger* and *Aspergillus parasiticus*. *Petrol. & Environ. Biotech.* **3,** 119-127 (2012).

7- Gochev, V.K., Velkova, Z.I, Stoytcheva, M.S. Hexavalent chromium removal by waste mycelium of *Aspergillus awamori*. *J. Serb. Chem. Soc.* **75,** 551-564 (2010).

8- Huang, H., Wu, K., Khan, A., Jiang, Y., Ling, Z., Liu, P., Chen, Y., Tao, X., Li, X. A novel *Pseudomonas gessardii* strain LZ-E simultaneously degrades naphthalene and reduces hexavalent chromium. *Bioresour. Technol*. **207**, 370-378 (2016).

9- Nepple, B.B., Kessi, J., Bachofen, R. Chromate reduction by *Rhodobacter sphaeroides*. *J. Ind. Microbiol. Biotechnol.* **25**, 198–203 (2000).
